# Refuge or Reservoir? The Potential Impacts of the Biofuel Crop *Miscanthus x giganteus* on a Major Pest of Maize

**DOI:** 10.1371/journal.pone.0008336

**Published:** 2009-12-16

**Authors:** Joseph L. Spencer, S. Raghu

**Affiliations:** 1 Illinois Natural History Survey, University of Illinois, Champaign, Illinois, United States of America; 2 Arid Zone Research Institute, Alice Springs, Northern Territory, Australia; University College London, United Kingdom

## Abstract

**Background:**

Interest in the cultivation of biomass crops like the C4 grass *Miscanthus x giganteus* (*Miscanthus)* is increasing as global demand for biofuel grows. In the US, *Miscanthus* is promoted as a crop well-suited to the Corn Belt where it could be cultivated on marginal land interposed with maize and soybean. Interactions (direct and indirect) of *Miscanthus*, maize, and the major Corn Belt pest of maize, the western corn rootworm, (*Diabrotica virgifera virgifera* LeConte, WCR) are unknown. Adding a perennial grass/biomass crop to this system is concerning since WCR is adapted to the continuous availability of its grass host, maize (*Zea mays*).

**Methodology/Principal Findings:**

In a greenhouse and field study, we investigated WCR development and oviposition on *Miscanthus*. The suitability of *Miscanthus* for WCR development varied across different WCR populations. Data trends indicate that WCR populations that express behavioural resistance to crop rotation performed as well on *Miscanthus* as on maize. Over the entire study, total adult WCR emergence from *Miscanthus* (212 WCR) was 29.6% of that from maize (717 WCR). Adult dry weight was 75–80% that of WCR from maize; female emergence patterns on *Miscanthus* were similar to females developing on maize. There was no difference in the mean no. of WCR eggs laid at the base of *Miscanthus* and maize in the field.

**Conclusions/Significance:**

Field oviposition and significant WCR emergence from *Miscanthus* raises many questions about the nature of likely interactions between *Miscanthus*, maize and WCR and the potential for *Miscanthus* to act as a refuge or reservoir for Corn Belt WCR. Responsible consideration of the benefits *and* risks associated with Corn Belt *Miscanthus* are critical to protecting an agroecosystem that we depend on for food, feed, and increasingly, fuel. Implications for European agroecosystems in which *Miscanthus* is being proposed are also discussed in light of the WCR's recent invasion into Europe.

## Introduction

The production of fuel from crop sources is gaining momentum globally; world ethanol production is expected to double between 2008 and 2017 [1]. While much of the current biofuel production is from grain/food crops (e.g. corn, sugarcane, soybean), there is considerable research investment in the development of biomass crops with the primary purpose of bioenergy production [2], [3]. To avoid competition in land-use for grain/food versus fuel crops, there are growing calls for the utilization of marginal lands (e.g. Conservation Reserve Program [CRP]) and lands of low production value for biomass crops [4]. The conversion of marginal lands into monoculture production areas for biomass crops is a topic of vigorous debate with various unresolved issues, notably the relative carbon sequestration benefits and productivity of monocultures vs. polycultures [5], the disruption of ecosystem services provided by marginal lands [6], [7], and the invasiveness risks of the species being considered [8], [9]. In addition to these issues, the direct and indirect effects [10] of biomass crops on other crops and pests in the agroecological landscape need to be carefully examined.

**Figure 1:**
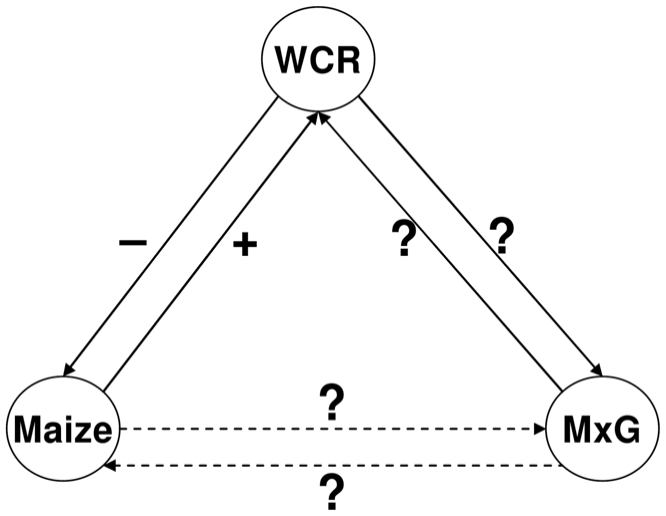
Potential interactions of *Miscanthus*, maize and western corn rootworm. Schematic representation of direct and indirect effects of introduction of the biomass grass-crop *Miscanthus* (MxG) into a landscape dominated by the food grass-crop maize. The direct effects (solid arrows) of *Miscanthus* on a maize pest species (western corn rootworm; WCR) and the resultant indirect effects (dashed arrows) of *Miscanthus* on corn are unknown.

In the Midwestern USA, the C_4_ grass *Miscanthus* x *giganteus* (*Miscanthus* hereafter) is actively promoted for biofuel production. This region is also part of the world's most productive and expansive maize-growing region, *ca*. 19% of the world's harvested corn hectares are found in 12 Corn Belt states [11], [12]. The introduction of a grass crop for biomass production (*Miscanthus*) in a landscape dominated by a grass, grain crop (maize) creates a potential for numerous indirect interactions between *Miscanthus* and corn. The exotic status of *Miscanthus* may be an advantage because of a lack of natural enemies that may otherwise limit its productivity [2], [13]; however, the potential for pests of native or crop species to utilize *Miscanthus*, and the resultant indirect effects of this biomass crop on corn production (Fig. 1) have not been adequately addressed. This is particularly important in light of the legacy of interactions between maize and its major pest, *Diabrotica virgifera virgifera* LeConte (western corn rootworm; WCR hereafter).

### Legacy of WCR – Maize Interactions

WCR is a destructive maize pest, responsible for in excess of US$1 billion in annual yield losses and control costs in the U.S. Corn Belt [14]. WCR biology is tied to maize [15]. Females of the univoltine WCR historically displayed a strong fidelity to cornfields for feeding and egg laying. Upon emergence from the overwintering eggs, WCR larvae must quickly locate host roots and feed or else they will die. WCR larval development is possible on maize roots and those from a number of grass species; while complete development to adults is possible only on the roots of maize and a subset of grasses supporting larval development [16]–[21]. Only where maize is grown in the same field in successive years (continuous maize) can WCR populations be sustained. The female fidelity to cornfields and larval dependence on maize roots underlie the long-recognized use of annual crop rotation as a WCR management tool. Annual rotation of maize with a non-host crop (e.g. soybean) eliminates the WCR threat because plants that do not support larval development are planted where WCR egg laying was focused the previous year (i.e. cornfields).

Recommended as a plainly obvious solution to rootworm problems since the 19^th^ Century [22], adoption of crop rotation has fluctuated depending on local/regional demand for maize, availability of irrigation, and adoption of other pest management alternatives. In recent decades, a primarily corn-soybean annual crop rotation provided excellent control of WCR and still dominates the Corn Belt [23]. Nationwide, approximately 80–82% of maize is grown in a rotation of some kind [24], [25]. Because crop rotation destroys the offspring of females with strict egg-laying fidelity to cornfields, it likely selected for females with reduced egg-laying fidelity to cornfields and increased mobility [26], [27]. Within 20 years of WCR arrival in Illinois, intensive (94–98%) adoption of cultural control for rootworm management has unwittingly changed a cornfield specialist into a pest whose egg-laying females could be collected from almost any crop in the landscape (including maize) [28], resulting in a behavioural resistance to crop rotation [29]. Since rotation resistance was first recognized in 1995, it has expanded from its Illinois-Indiana epicenter into Michigan, Ohio, Wisconsin, Iowa, and Ontario, Canada [30], leading to an increased reliance on chemical control methods.

In a regulatory climate where insecticide was replacing the use of an environmentally benign management tool, development and commercialization of rootworm-resistant *Bt* transgenic (TG) maize hybrids [31] was highly anticipated. Like previously available *Bt* maize hybrids targeting lepidopteran pests, producers using TG hybrids for rootworm management were required to reserve 20% of each TG cornfield for planting a hybrid that did not express the rootworm-killing *Bt* toxin present elsewhere. This non-TG ‘refuge’ allows a large population of WCR larvae to develop without any exposure to *Bt* toxin. When the large population of *Bt*-susceptible refuge WCR emerge as adults, the mate-seeking males disperse into the more sparsely inhabited TG portions of a field. In TG maize, refuge males will greatly out-number any potentially-resistant TG-field survivors and secure most of the matings with TG-field females. Diluting the TG-field population with refuge beetles (‘refuge strategy’), reduces the likelihood that two individuals carrying genes for resistance will mate, thus slowing the rate of resistance development [32]. The risk of WCR resistance to TG maize is significant; in a pair of recent greenhouse studies WCR populations reared without a refuge developed resistance rapidly to TG maize hybrids [33], [34]. The importance of using refuges as part of insect resistance management (IRM) for WCR is growing as adoption of TG hybrids increases and fewer conventionally managed acres of non-TG corn remain to serve as ‘natural’ refuges. In 2008, 52% of Illinois maize was an insect-protected transgenic corn hybrid [35]. The next chapter of WCR-maize interactions will be written in an atmosphere where the threat of WCR resistance to TG maize will loom large. The presence of perennial biofuel grasses like *Miscanthus* in Midwestern US agroecosystems may alter historical patterns of pest-host interaction. This has significant implications beyond U.S. agroecosystems; WCR has been detected in Europe since 1992 and there the expanding population is under intense management as an invasive pest [36], and *Miscanthus* is among the most widely promoted perennial biomass crops in Europe [37].

The potential agronomic and ecological consequences of introducing biofuel grasses into Midwestern agroecosystems and elsewhere have not received adequate attention. In particular, the direct effects of *Miscanthus* on WCR (solid arrows in Fig. 1) and the resultant indirect effects of *Miscanthus* on maize (dashed arrows in Fig. 1) are unknown. It is vital to understand these interactions as they have the potential to (a) influence maize yields (through apparent competition between maize and *Miscanthus* mediated by WCR) and, (b) influence IRM and the utility of rootworm-resistant transgenic (TG) corn hybrids. Understanding these effects will also improve our ability to comprehensively evaluate the risks and benefits of large-scale deployment of a biomass grass-crop in a food grass-crop dominated agricultural landscape.

The ability of *Miscanthus* to support the development of WCR is the subject of this investigation. Specifically, we address the following questions.

- Relative to maize, what is the ability of *Miscanthus* to support larval development of WCR?
- Does *Miscanthus*' suitability as a host change across genetically distinct WCR populations?
- Does WCR lay eggs under *Miscanthus* under field conditions?

## Materials and Methods

### Plants

Maize cultivar FR1041 x FR697 was used for Experiments 1 and 2; Maize cultivar B73 x M017 was used for Experiments 3 and 4 (Tables 1, 2). Maize was planted 2.5 cm deep into a central 15 cm ×15 cm core of topsoil surrounded by a potting soil and sand mixture in 30 cm diameter pots. A slow-release fertilizer (Osmocote®, The Scotts Company LLC, Marysville, OH 43041, USA) was added to pots after planting. Plants were held in greenhouse rooms at 25°C under natural light with auxiliary daylight-balanced fluorescent lighting at night.

**Table 1:** Origin and life-history traits of *Diabrotica virgifera virgifera* (WCR) populations used in this study.

WCR populations	Origin	Year of field-collection^a^	Diapause (Y/N)	Rotation resistant (Y/N)^b^
1	Brookings Co., SD	1966	N	N
2	Moody Co., SD	1986	Y	N
3	Benton Co., IN	2001	Y	Y^c^
4	Champaign Co., IL	2007	Y	Y^d^

a WCR colonies were maintained in laboratory after field collection, prior to use in this study.

b Rotation resistance refers to the possession of behavioral traits of reduced fidelity to laying eggs in cornfields and greater mobility.

c Initial eggs obtained from females collected as adults from soybean fields.

d Eggs obtained from females collected as larvae from first-year (rotated) cornfields.

**Table 2:** Design of experiments undertaken to evaluate the relative suitability of different maize cultivars and *Miscanthus* x *giganteus* as hosts for *Diabrotica virgifera virgifera* (WCR).

Experiment^a^	Maize cultivar	WCR populations^b^	WCR eggs/plant
1	FR1041 x FR697	1	100
2	FR1041 x FR697	1	100
3	B73 x M017	1	100
	B73 x M017	2	100
	B73 x M017	3	100
	B73 x M017	4	50
4	B73 x M017	1	100
	B73 x M017	2	100
	B73 x M017	3	100
	B73 x M017	4	50

a Replication dictated by availability of eggs from different WCR populations. Replication for Experiments 1, 2 and 3 were 5, 13 and 15 respectively. For Experiment 4, replications levels were 7–12 replicates for maize and 4–5 replicates for *Miscanthus*.

b See Table 1 for characteristics of each population.

*Miscanthus* rhizomes were obtained from Steve Schmidt Nursery, (Eagle Creek, OR 97022, USA) and grown in 20 cm diameter pots in a potting mix (2 parts of peat [Sun Gro Horticulture, Bellevue, WA 98008, USA], 1 part of Perlite [Midwest Perlite Inc., Appleton, WI 54913–823, USA], 1 part of Vermiculite [Therm-O-Rock East, Inc. New Eagle, PA 15067, USA]) amended with ECO*pH*RST™ dolomitic lime [1.8 kg/m^3^; National Lime and Stone Company, Findlay, OH 45840 USA]). At the time of maize planting for Experiments 1 and 2, 1.0 m tall *Miscanthus* was transplanted into the centers of 30 cm diameter pots, and ringed with a 15 cm deep layer of topsoil. For Experiments 3 and 4, the root mass of *Miscanthus* from 30 cm diameter pots was divided into quarters with a reciprocating saw and transplanted as described for *Miscanthus* rhizomes.

An artificial potting mix was used in this study rather than field soil to minimize the risk of contamination with WCR eggs from field soil. All plants were watered daily (2 L/day) with a drip irrigation system.

### Insects

Four populations of WCR (Coleoptera: Chrysomelidae) with various diapause requirements were used in this study (Table 1). In addition, two of these populations were collected from areas where WCR exhibit behavioural resistance to crop rotation (Table 1), a trait characterized by a reduced egg-laying fidelity to cornfields and greater mobility [23], [29], [38]. These behaviourally-variant populations occur across the Midwestern Corn Belt and across the intended cultivation range of *Miscanthus* in the USA [30].

WCR populations 1–3 were sourced from colonies maintained at the USDA-ARS North Central Agricultural Research Laboratory in Brookings, SD. Eggs were shipped on fine soil and held at 10°C until 2–3 weeks before the start of the experiments. Populations 2 and 3 were shipped after egg diapause completion, eggs from the nondiapause population hatch *ca*. 2 weeks after they are laid. For population 4, eggs were collected from WCR adults that emerged from the roots of first-year-maize at the University of Illinois, Shaw Farm (Urbana, IL). Eggs of this population were held for two-three weeks at room temperature after oviposition and then transferred into dishes of moist gravel and stored at 10°C for 17 weeks to complete egg diapause.

Prior to the start of the experiments, eggs were washed from the storage media and held at room temperature in shallow containers of tap water until evidence of larval development was revealed by yolk condensation in a majority of eggs. Developing eggs from populations 1–3 were counted and pipetted as groups of 100 into glass vials with a small volume of water on the day before an experiment began. Because eggs from Champaign Co., IL (population 4) were limited, they were dispensed to vials in groups of 50 eggs. These egg densities are well below the level (500 eggs/plant) at which any density-dependent effects (e.g. competition for root resources, cannibalism) have been detected [39].

### Greenhouse Experiments

Experiments were carried out in the greenhouses used for growing the plants. Using a factorial design (Table 2), each plant was inoculated with WCR eggs from different populations 25–30 d after planting (maize plants were in V6–V8 stage). A 2–4 cm deep opening was made in the soil 2.5 cm away from the base of a maize plant or the edge of a *Miscanthus* rhizome mass. The WCR eggs were dispensed by pipetting the contents of an egg vial into the bottom of the opening and then closing the hole. Once treated, 100 cm tall conical wire garden frames were inserted into each pot around the plant and a 250 cm tall fine mesh fabric bag (with a drawstring closure) was slipped over the frame and secured around the top of the plastic pot. The treated plants were distributed randomly on greenhouse benches.

Beginning three weeks after inoculation, plants were monitored each morning for emergence of adult WCR. Newly-emerged adults were collected and identified to sex before they were frozen at −80°C. Daily inspection continued until 7 days passed without collection of an adult from any plant. The total duration of each of the experiments ranged from 4–6 weeks. After completion of each experiment, the WCR were dried in individual 2 ml microcentrifuge tubes for 1 week at 70°C and adult dry weight was recorded.

### Oviposition by WCR in Maize vs. *Miscanthus* in the Field

We examined the likelihood of WCR oviposition around *Miscanthus* and maize, under field conditions. On July 24, 2007, sixteen *ca*. 2 m tall clumps of *Miscanthus* in 30 cm dia. pots were buried as two rows of 8 plants into two soil trenches separated by 0.76 m along the west side of a 1.6 ha cornfield. The trenches occupied the former location of two rows of maize that had been removed from the rows several weeks earlier. After placement, the trench was filled and the soil level was groomed to match the rims of the buried pots. The plants were present in the field through peak WCR flight and remained in place until the week before maize harvest. An on-site weather station recorded that 13.3 cm of rainfall fell during the period of the field experiment; supplemental water was provided to the *Miscanthus* and adjacent two rows of maize plants once. To facilitate pot removal, on September 12, the *Miscanthus* foliage was clipped to within 10 cm of the soil surface, steam sterilized, and discarded. WCR adults were no longer present in the field when these maize and *Miscanthus* plants were removed.

On September 28, 2007, seven soil cores (10 cm diameter x 10 cm depth) were collected from between the two rows of buried *Miscanthus* pots, between the interior *Miscanthus* row and the adjacent row of maize, and between the adjacent maize row and its neighboring maize row. In addition, eight soil cores were also collected near the bases of eight maize plants in each of the two maize rows; the selected maize plants were those closest to the *Miscanthus* clump in the adjacent row. Each soil sample was mixed before a 0.5 L portion was removed and stored in a plastic bag at 10°C. After sampling the soil near maize plants and between *Miscanthus* rows, the buried *Miscanthus* pots were lifted from the soil and returned to the laboratory. Soil samples from *Miscanthus* plants were collected by combining the *ca*. 1 cm thick layer of loose soil from surface of each pot with the outside 3–5 cm wide ring of soil surrounding the root mass to a depth of 10 cm (the dense mass of fleshy rhizomes prevented sampling from within the root mass). A 0.5 L sample was removed and stored as described above. After soil sampling, the *Miscanthus* plants were steam sterilized and discarded.

Using the method of Shaw et al. [40], each soil sample was washed and the eggs were recovered. Eggs were inspected and counted under a stereo microscope and identified to species based on chorionic sculpturing using a compound microscope [41].

### Data Analysis

For the greenhouse experiments, a factorial ANOVA was used to examine the relative suitability of maize and *Miscanthus* for WCR development (Table 2). An initial two-way ANOVA on the combined data from Experiments 1 and 2 was done with experiment and host as factors. Any differences between hosts were consistent across experiments (i.e. no host*experiment interaction effect) and so data from Experiments 1 and 2 were combined for analysis using a one-way ANOVA with host species as the factor. Similarly an initial 3-way ANOVA with experiment, host and egg source as factors revealed that any differences between hosts or egg sources were consistent across experiments (i.e. no host*egg source*experiment interaction effect). Hence data from Experiments 3 and 4 were combined for analysis using a two-way analysis with host species and WCR egg source as factors in the analysis. Response variables included overall adult emergence (as a % of the eggs inoculated) and adult dry weight. Emergence patterns over time were graphically examined.

Differences in the number of eggs laid by WCR in the field at the base of *Miscanthus* plants, maize plants, or the inter-row space between maize and *Miscanthus* were examined using a factorial ANOVA.

Tukey's HSD test was used for post-hoc pairwise comparisons. All analyses were done using SYSTAT 8.0.

## Results

### Experiments 1 and 2

**Figure 2:**
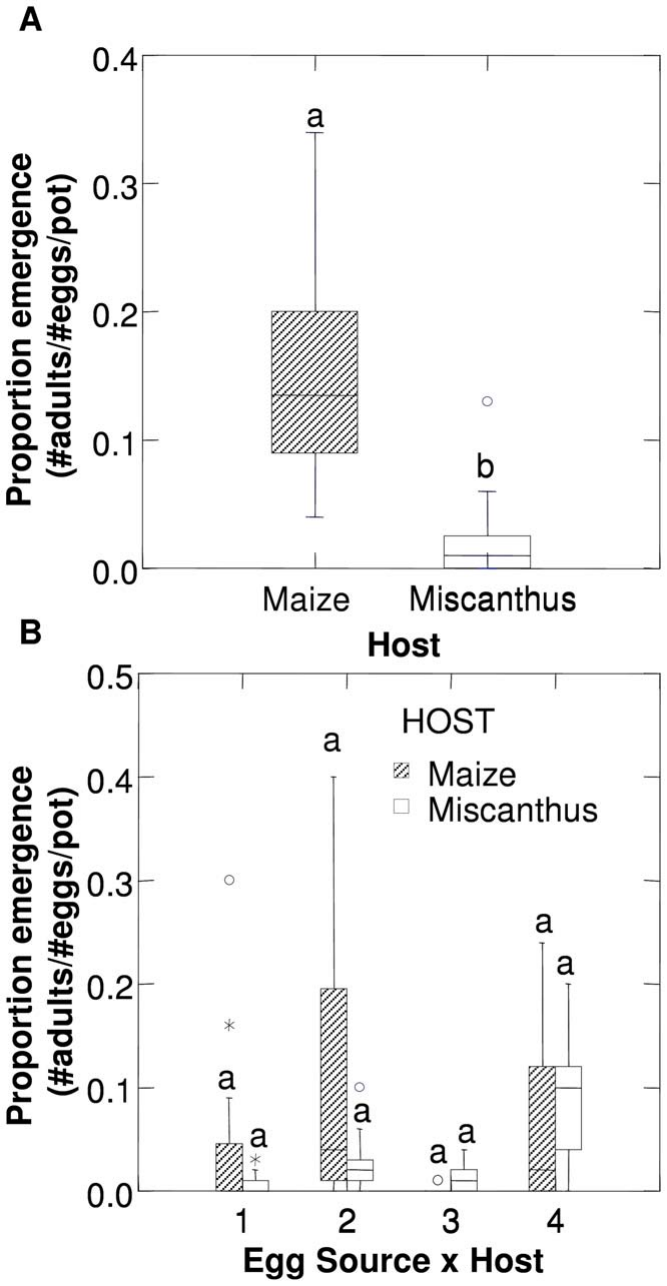
Relative development of western corn rootworm populations on maize and *Miscanthus*. (A) Proportional emergence of adults of the non-diapausing Brookings (South Dakota) western corn rootworm (WCR) population (Experiments 1 & 2). (B) Proportional emergence of adults from WCR populations that differ in their diapause characteristics and rotation-resistance status (Experiments 3 & 4). Lettering above box-plots indicates posthoc pairwise comparison of means as revelaed by a Tukey's HSD test. See Table 1 for details of WCR population characteristics.

The proportion emergence of WCR adults from maize was greater (F_1,34_ = 33.22, P<0.001; Fig. 2A) than *Miscanthus* for the non-diapausing Brookings (South Dakota) population of WCR. Significantly more male WCR (F_1,34_ = 25.11, P<0.001), female WCR (F_1,34_ = 28.84, P<0.001), and total WCR (F_1,34_ = 33.22, P<0.001) emerged from maize than *Miscanthus*. Adult WCR emergence from *Miscanthus* (38 WCR) was 13.8% of that from maize (276 WCR).

**Figure 3:**
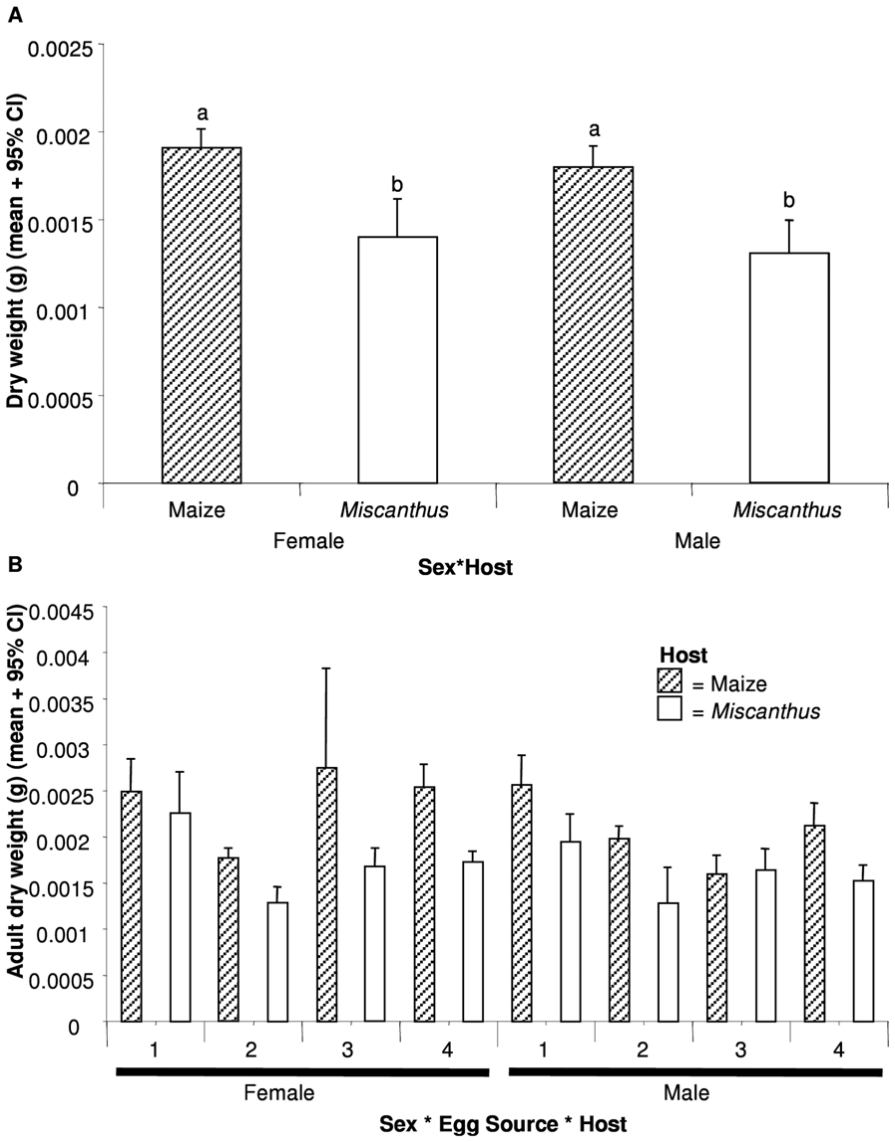
Dry weight (g) of western corn rootworm adults developing on maize and *Miscanthus*. (A) Weight of adults of the non-diapausing Brookings (South Dakota) western corn rootworm (WCR) population (Experiments 1 & 2). (B) Weight of adults from WCR populations that differ in their diapause characteristics and rotation-resistance status (Experiments 3 & 4). Bars depict means and error bars are 95% confidence intervals. Lettering indicates posthoc pairwise comparison of means as revealed by a Tukey's HSD test. See Table 1 for details of WCR population characteristics.

Host plant had a significant effect on dry weight of WCR (F_1,285_ = 17.791, P<0.001) with adult beetles emerging from corn being heavier than those from *Miscanthus* (Fig. 3A). The sexes did not differ in their weight and there were no host * sex interaction effects (Fig. 3A).

### Experiments 3 and 4

There was no difference in the proportional emergence of WCR between maize and *Miscanthus*. The significant interaction effect (egg source*host: F_3,173_ = 6.52, P<0.001) is the result of variability in the proportion of WCR emerging from different egg sources (egg source: F_3,173_ = 14.97, P<0.001). The trends in the data suggest that the rotation-resistant populations perform better on *Miscanthus* than maize (Fig. 2B).

There was an effect of host and WCR source population (host: F_1,173_ = 13.51, P<0.001; egg source: F_3,173_ = 8.89, P<0.001) on the number of male WCR, but these effects were not independent of each other as evident from the significant interaction effect (egg source*host: F_3,173_ = 8.51, P<0.001). More males were produced from maize than *Miscanthus* in the Moody Co. diapausing population. For the other three populations, there was no difference in the number of males emerging from maize and *Miscanthus*.

There was no difference in the number of female WCR emerging from maize and *Miscanthus*. The significant interaction effect (egg source*host: F_3,173_ = 5.13, P = 0.002) was the result of variability in the number females emerging from different egg sources (egg source: F_1,173_ = 8.89, P<0.001).

There was an effect of host on total WCR, but this varied in relation to egg source as evident from a significant interaction effect (egg source*host: F_3,173_ = 6.48, P<0.001). More WCR were produced from maize than *Miscanthus* in the Moody Co. diapausing population. For the other three populations, there was no difference in the number of males emerging from maize and *Miscanthus*. Adult WCR emergence from *Miscanthus* (174 WCR) was 39.5% of that from maize (441 WCR).

A 3-way ANOVA revealed that egg source (F_3, 626_ = 8.742, P<0.001) and host (F_1,626_ = 14.880, P<0.001) affected WCR dry weight independent of each other and sex (i.e. none of the 2- or 3-way interaction terms were statistically significant). Eggs developing on *Miscanthus* resulted in adults that were 75–80% of the weight of those developing on maize (Fig. 3B). A Tukey's HSD test revealed that eggs from Champaign Co., IL and Brookings Co., SD WCR populations yielded heavier adults than those from the Moody Co., SD WCR population.

### Relative Rates of Emergence of WCR from Maize vs. *Miscanthus*

**Figure 4:**
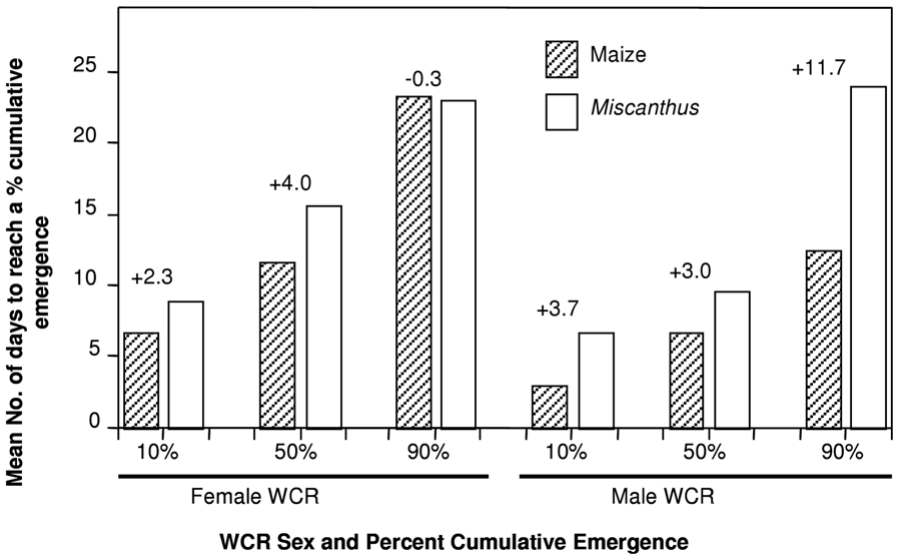
Relative rates of emergence of male and female western corn rootworm developing on maize and *Miscanthus*. Numbers above bars are the difference between the average time taken by western corn rootworm (WCR) to reach a level of emergence on maize vs. *Miscanthus*.

WCR developing on maize emerged more rapidly than those developing on *Miscanthus* (Fig. 4). However, the rates of emergence for female WCR were similar by the time 90% of the adults had emerged (Fig. 4).

### Oviposition by WCR in Maize vs. *Miscanthus* in the Field

**Figure 5:**
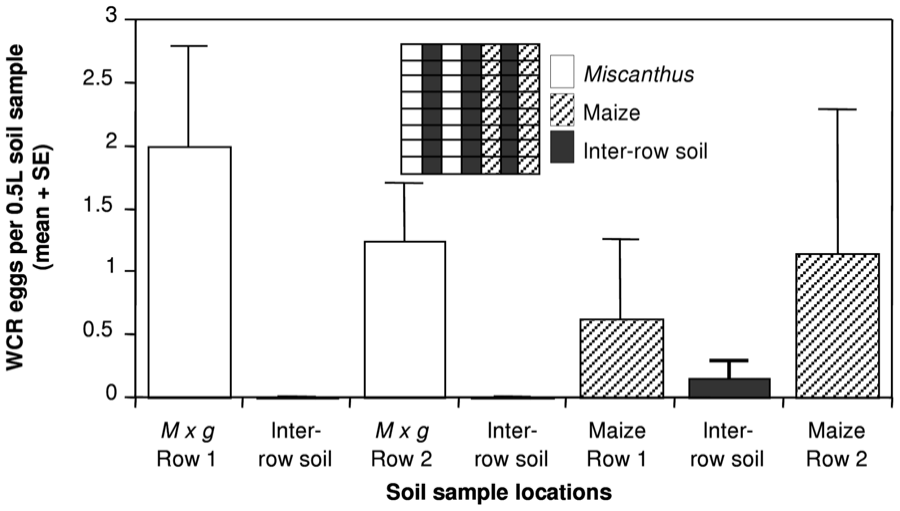
Relative oviposition by western corn rootworm in maize and *Miscanthus* under field conditions. Studies were undertaken in Champaign Co., IL. Inset depicts layout of plants in the field.

There was no difference in the number of eggs laid by field WCR under maize and *Miscanthus* (F_6,45_ = 1.59, P = 0.17; Fig. 5). More eggs were laid around plants than in the inter-row spaces (Fig. 5).

## Discussion

A lack of susceptibility to pests is among the cited agronomic and economic qualities of *Miscanthus* favoring its cultivation as a biomass/biofuel crop [37]; insecticide inputs are not included in economic analyses used to demonstrate economic viability of *Miscanthus* (e.g. [42]). Our results demonstrate that the most significant U.S. pest of maize can complete development on *Miscanthus*, and that at least one of its genotypes (i.e. Illinois rotation-resistant population) will lay eggs around *Miscanthus* in the field. The lower proportion emergence (*ca.* 30%) and the reduced weight (*ca.* 75–80%) of adults emerging from maize relative to *Miscanthus* in this study, is similar to WCR reared on grasses regarded as alternate WCR hosts [20]. The ecological and economic implications of these results need careful examination to elucidate the role *Miscanthus* may play in mediating WCR-maize interactions.

The impacts of WCR as a pest on *Miscanthus* might not have a significant effect on biomass yield owing to the perennial nature of the abundant roots. While this might be an advantage of *Miscanthus* production, it is naïve to expect that Corn Belt pest ecology will be unaltered (as is often assumed in economic models of biofuel production, e.g. [42]) when a demonstrated perennial host (*Miscanthus*) of an adaptable and economically important maize pest is added to the system. Because there are no commercial-scale data on direct and indirect interactions between WCR, maize and *Miscanthus*, we investigated the direct effects of *Miscanthus* on WCR relative to maize. Based on our results, combined with literature on WCR ecology and behaviour, we present below a range of plausible/probable positive, negative, direct and indirect effects to maize production that may arise from production scale cultivation of Corn Belt *Miscanthus*.

### *Miscanthus* – Refuge or Reservoir?

An increasing proportion of maize grown in the U.S. is TG maize. The presence of a perennial host of WCR in such an environment could be beneficial if *Miscanthus* can act as a refuge from a resistance management perspective. The potential for interactions may be most acute where rotation-resistant WCR populations are present; the lack of ovipositional host fidelity in these populations [38] makes it likely that *Miscanthus* plantings will be the target of significant egg-laying. Whether *Miscanthus* acts as a refuge depends on how easily WCR individuals might be able to move/disperse through the dense vegetation. If WCR's abilities to enter and leave *Miscanthus* are poor, WCR populations would be spatially isolated from those originating in maize and experience selection for a WCR population better adapted to development on *Miscanthus*. As is evident from our results, there is sufficient variability among the populations we used to illustrate WCR development on *Miscanthus* for such selection to be plausible. Poor movement/dispersal might also be a benefit by favouring WCR with strong host fidelity, and if there is genetic variation for this trait then selection may act against behaviours that facilitate rotation resistance.

The utility of *Miscanthus* as a refuge will also be dictated by the level of synchrony in phenology of WCR biology in *Miscanthus* and maize. WCR emergence is more closely synchronized between maize and *Miscanthus* than a number of grasses considered alternate hosts [43], [44], suggesting there is the potential for populations developing on these two hosts to interact during the mating period. If realized, such population mixing would benefit the cause of IRM by facilitating mating between TG-susceptible WCR from *Miscanthus* and any potentially-resistant individuals emerging from TG maize.

While the potential for *Miscanthus* to serve as a refuge is alluring, there is sufficient cautionary evidence that its projected distribution throughout the Corn Belt might also cause problems. Historically, it was the reliable availability of rootworm hosts between years (e.g. cultivation of continuous maize) that allowed rootworm beetles to become pests [30], [45]. In 2008 the total area under maize in the U.S. was 34.4×10^6^ha [46], since 18–20% of US maize is not grown in a rotation [24], [25], ca. 7.0×10^6^ha of continuous maize was planted. *Miscanthus*' suitability as a host for WCR makes it ecologically equivalent to continuous maize. Therefore, the proposed addition of 7.9×10^6^ha of *Miscanthus* plantings in the Corn Belt [3] would more than double (*ca*. 113%) the acreage of continuous maize equivalents. Even accounting for the slightly diminished development of WCR from *Miscanthus* relative to maize, our results suggest that *Miscanthus* could function as a reservoir enhancing pest pressure on maize by enabling the build-up of WCR populations. The distance between the planned *Miscanthus* acreage (including those on CRP lands) and current corn acreage are well within typical movement and dispersal capacity of this highly mobile insect species [26], [28]. While this represents a significant risk in the Corn Belt of the U.S., perhaps the risk is even greater in Europe where invading WCR populations are still expanding into new areas [47]. Because European growers rely on crop rotation for WCR management [30], foliar and soil insecticides are rarely used [48] and the adoption of TG maize is in its infancy, they are more vulnerable to mounting populations. This risk will only be accentuated if *Miscanthus*, which is among the most widely promoted biomass crops in Europe [37], can function as a reservoir for WCR.

The likelihood of *Miscanthus* functioning as a reservoir for WCR is not altogether unrealistic; the closely related *Miscanthus sinensis*, a hypothesized parent species of *Miscanthus*, is known to mediate interactions between crops and their pests in its native range [49]. The potential for *Miscanthus* to serve as a reservoir presents several significant challenges to the management of WCR. How might pest WCR be treated within dense *Miscanthus* growth or would maize need to be sprayed with pesticides to manage the WCR populations that develop on *Miscanthus* and move into cornfields? How will the cost of such management affect the economic viability of *Miscanthus* as a biofuel crop?

Current economic models of the agronomic viability of *Miscanthus* (e.g. [42]) do not factor in any costs for pest management. Excluding plausible or predictable pest interactions from risk-cost-benefit analyses handicap efforts to reliability model and compare bioenergy costs. As an example, annual pesticide inputs ($98.84/ha) in central Illinois maize systems accounted for 14% of per hectare non-land costs between 2003–2007 [50], [51]. Assuming a similar unit pesticide expense in *Miscanthus*, based on projected 2009 budgets for maize ($112.00/ha) WCR management costs would add 11% to the projected annualized operating cost calculated by Khanna et al. [42] and change the break-even costs for bioenergy.

Uncertainty about potential costs and/or benefits of Corn Belt *Miscanthus* must also be weighed relative to the value of annual U.S. maize production ($52 billion in 2007; [46]). The impact of a *Miscanthus*-associated increase in WCR pest activity would be multiplied due to the vast scale of the proposed interactions. Projections for the cost of such externalities are difficult to know because these risks have not been part of the conversation thus far about Corn Belt *Miscanthus*. It is sobering to consider what it might cost to insure against such risks, and how the costs of such insurance might influence the agronomic viability of *Miscanthus*.

WCR development on *Miscanthus* cautions against uncritical acceptance of the veracity of the claims of its suitability for the U.S. maize-growing regions (e.g. [13]). We acknowledge that the Corn Belt agricultural ecosystem is vastly more complex than any greenhouse or fieldplot; clearly, it would be ill-advised to predict a particular trajectory for WCR-*Miscanthus*-maize interactions based solely on our findings. However, in a system that is relied upon for feed, food, and increasingly fuel, it seems only rational to thoroughly assess the prospect for *Miscanthus* to affect the ecology of the system's most significant pest prior to establishment of large monocultures. This study is a first step in this regard.
